# Dupuytren-Like Contracture of the Foot: Ledderhose Disease

**DOI:** 10.1055/s-0036-1593355

**Published:** 2016-09-21

**Authors:** Osman Akdag, Gokce Yildiran, Mehtap Karamese, Zekeriya Tosun

**Affiliations:** 1 Department of Plastic, Reconstructive, and Aesthetic Surgery, Selcuk University Medical Faculty, Konya, Turkey

**Keywords:** Dupuytren, foot, Ledderhose disease, plantar fibromatosis

## Abstract

**Introduction** Plantar fibromatosis is a rare hyperproliferative disease of plantar aponeurosis and is also called Ledderhose disease. Case properties and treatment are discussed in this report.

**Case Report** A 30-year-old man presented with painful bilateral plantar nodules. He had multiple and bilateral fixed and solid nodules on the plantar and medial side of his feet measuring 1 cm each. Ultrasound was performed and hypoechoic homogeneous nodules were detected. The patient underwent surgery, and the nodes were removed via a plantar incision with 2-cm safety distance.

**Discussion** Ledderhose disease is a rare, hyperproliferative disorder of the plantar aponeurosis. The nodules are slow growing and found in the medial part of the plantar fascia. The precise etiology remains unknown. The treatment options are conservative management, steroid injections, radiotherapy, and surgery.

**Conclusion** The main cause of this disease remains uncertain. Related conditions should be evaluated, and a patient who presents with Dupuytren or Peyronie disease should also be investigated for Ledderhose disease.

Plantar fibromatosis, or Ledderhose disease, is a rare hyperproliferative disease of plantar aponeurosis. 1 Georg Ledderhose described the disease in 1894 as a Dupuytren-like disease of the foot. 2 Nodules measuring 1 to 2 cm on the medial side of the foot arch are evidence for the disease. 3 Treatment of this disease is discussed via one case of plantar fibromatosis.

## Case Report

**Fig. 1:**
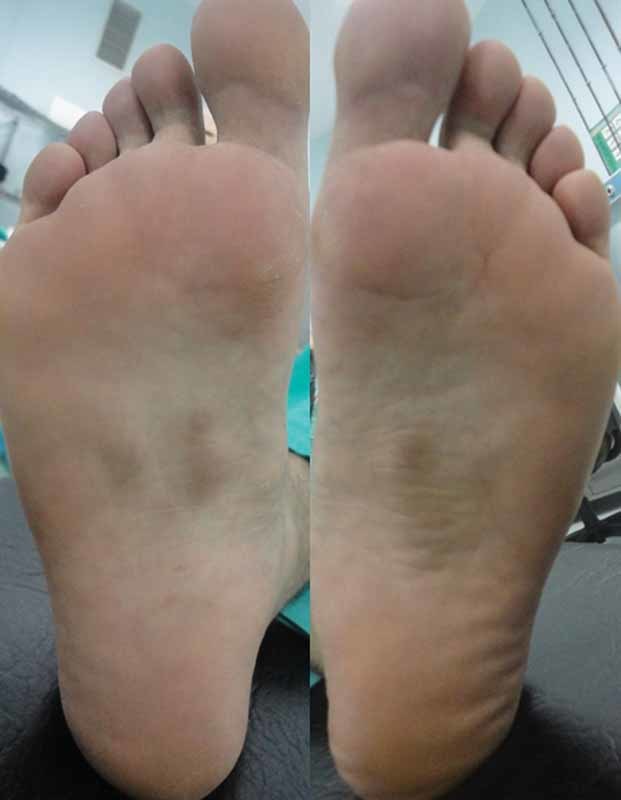
Preoperative view of nodules of plantar fascia.

**Fig. 2:**
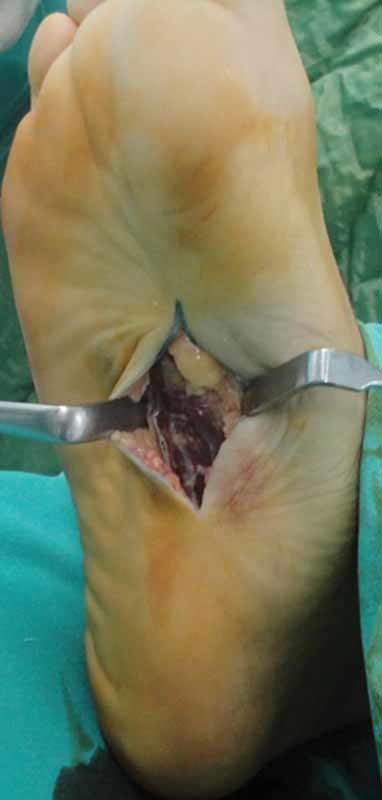
Intraoperative view of nodule.

**Fig. 3:**
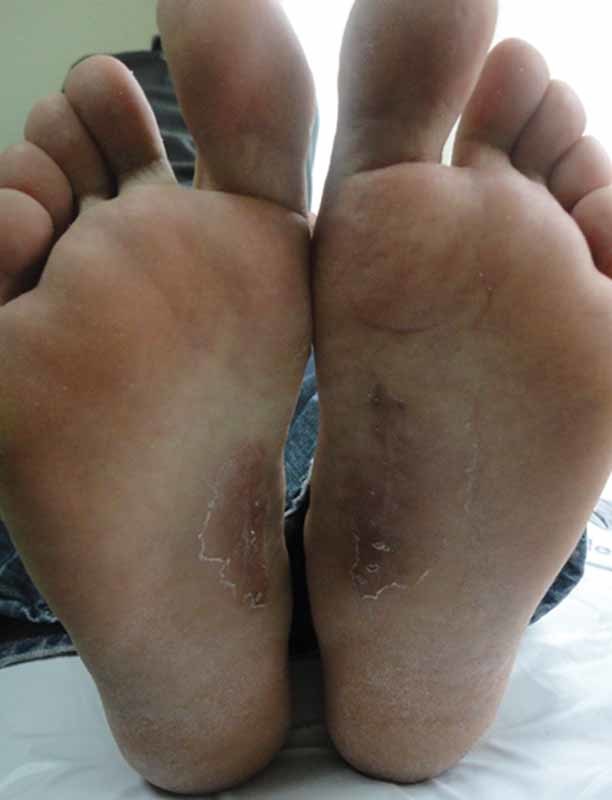
View at postoperative 8 weeks.

A 30-year-old man presented painful bilateral plantar nodules. Physical examination showed multiple and bilateral fixed and solid nodules measuring 1 cm each on the plantar and medial side of his feet ( Fig. 1 ). Five years earlier, the first painless nodule appeared in his right foot, and the number of nodules increased and become painful over the following years. He had using orthopedic insoles for the last 6 months. He was not taking any medication, and there was no family history of those nodules. Routine laboratory tests and blood glucose levels were normal. He did not have Dupuytren disease, diabetes mellitus, alcohol addiction, penile fibromatosis, epilepsy, or frozen shoulder. Ultrasound was performed, and hypoechoic homogeneous nodules were detected. The patient underwent surgery, and the nodes were removed via a plantar incision with 2-cm safety distance ( Fig. 2 ). There was no skin or muscle infiltration. The skin was closured primarily, wound dressing was changed once a day, and touching the ground was banned for 1 day. We continue to follow the patient ( Fig. 3 ).

## Discussion

Ledderhose disease is a rare, hyperproliferative disorder of the plantar aponeurosis. 1 4 The nodules are slow growing and found in the medial part of the plantar fascia. This nodular tissue is progressive and replaces normal aponeurosis. 2 The frequency of the disease was reported as 1.75/100,000 by Pickren et al and 1/100,000 by de Bree et al. 5 6 The disease is concomitant with Dupuytren disease in 2 to 18% and with Peyronie disease in 4% of patients. 7 8 Our patient had neither Dupuytren nor Peyronie disease. Males are affected more often than females, and symptoms usually start in the third to fourth decades. 1 Our case was a 30-year-old man. Ledderhose disease exhibits bilateral affection in 25% of cases, as in our patient. 9

The precise etiology remains unknown 1 2 10 11 ; however it is related with some conditions and diseases like Dupuytren disease, 7 frozen shoulder, alcohol addiction, epilepsy, diabetes mellitus, and penile fibromatosis. 10 11 In our case, none of these conditions existed and blood glucose levels were normal; the independence of our case from the known related conditions makes the case interesting.

The diagnosis of Ledderhose disease is usually clinical and does not frequently require confirmation. 12 However, Omor et al reported that magnetic resonance imaging has an important role in diagnosis and evaluating the severity of disease. 10 The differential diagnosis of plantar masses includes plantar fasciitis, leiomyoma, rhabdomyosarcoma, and liposarcoma. 10 We performed ultrasound to confirm the clinical diagnosis. After excision, histopathologic analysis revealed fibromatosis.

The treatment options are conservative management, steroid injections, radiotherapy, and surgery. Conservative treatment and steroid injections are options for painless and early painful stages. 11 However, the node's reduction or further relapses after healing are inevitable with only conservative therapies and without surgery. 11 13 14 15

When conservative treatments fail to reduce the symptoms, surgery is indicated. 11 Our patient used orthopedic insoles for the painful nodules for 6 months; however, because the symptoms were aggravated, surgical excision was indicated. Surgical excision is divided into three types: local, wide, and complete fasciectomy. 11 16 17 Van der Veer et al reported that local resections relapse with a percentage of 100% and complete fasciectomy relapses 25%. 18 Dürr et al reported that wide excision, with a safe distance of 2 to 3 cm, may relapse with a percentage of 78%. 19 Total removal of the plantar fascia results in extension of the foot longitudinal arch, so we performed a wide excision with a 2-cm margin.

## Conclusion

Plantar fibromatosis is rare, benign, easy to diagnose, hyperproliferative disease. However, the main cause of this disease remains uncertain, and related conditions should be evaluated. A patient who presents with Dupuytren or Peyronie disease should be investigated for Ledderhose disease. Further investigations are needed for certain etiopathogenesis of this rare condition.
